# Supplementary material for: Imaging Phenotypes and Evolution of Hepatic Langerhans Cell Histiocytosis on CT/MRI: A Retrospective Study of Clinical Cases and Literature Review
Source: Bioengineering (Basel). 2023 May 16;10(5):598. doi: 10.3390/bioengineering10050598 (PMC10215899; doi:10.3390/bioengineering10050598)
Supplement: Supplementary file 1 [file bioengineering-10-00598-s001.zip › bioengineering-2356572-supplementary.pdf]

Supplementary Material

Imaging phenotypes and evolution of hepatic Langerhans cell histiocytosis on CT/MRI: A retrospective study of clinical cases and literature review

*** Correspondence:**
Name: Yaqi Shen
Address: 1095 Jiefang Avenue, Qiaokou District, Wuhan, Hubei, China
Email: yqshen@hust.edu.cn.

1. Supplementary Figures

Figure S1

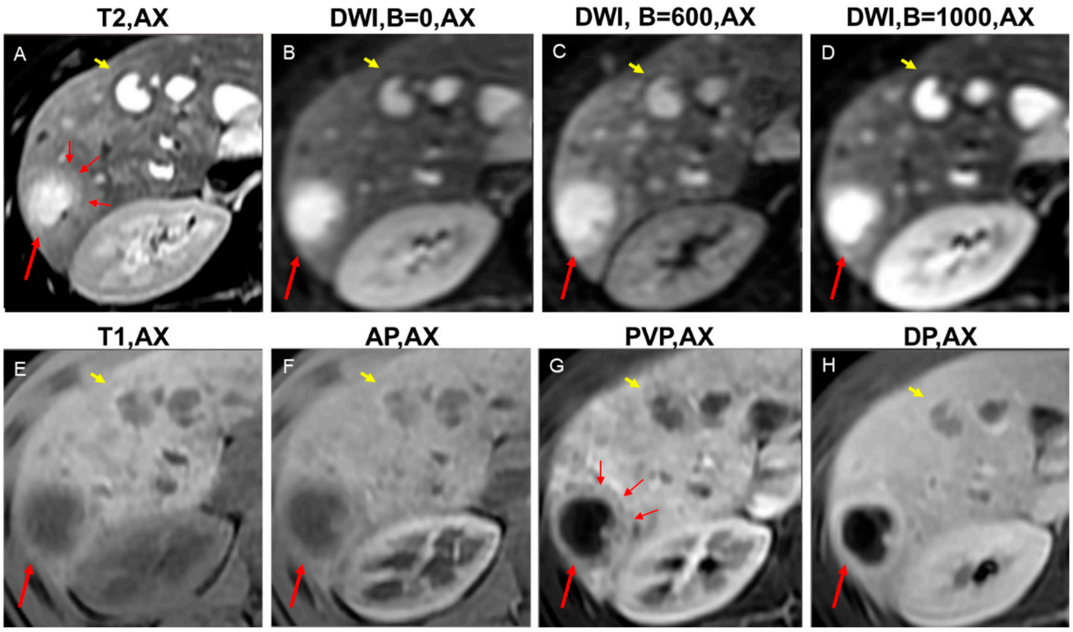

Figure S1. Imaging features of three kinds of parenchymal lesions

A 20-year-old man presented with abdominal pain for 20 days. He was diagnosed with multisystem LCH (MS-LCH) and classified as a disseminated lesion phenotype. MRI scans showing irregular, patchy, and miliary lesions (proliferative lesions) with thick-walled, cystic-like lesions (red arrow) (new granulomatous lesions), and thin-walled, cystic-like lesions (yellow arrow) (old granulomatous lesions). The thick and uneven

wall presented slight hyperintensity on T2WI, and miliary nodules (tiny red arrow) appearing higher/increased-intensity were occasionally seen in the thick wall on T2WI/DWI, and the intra-cyst appeared hyperintense on T2WI/DWI (A, B, C, D). Annular hypointensity on T2WI/DWI around the thin-walled cystic-like lesion was observed. Lesions presented hypointensity on T1WI (E). After administration of the contrast media, the patchy and miliary lesions showed mild-to-moderate enhancement, whereas the cystic-like lesions showed ring-like enhancement from the arterial phase to the delayed phase (F, G, H). Enhancement of the lesion relative to the background liver was most pronounced in the portal venous phase. Persistent enhancement and the extent of enhancement expand in delayed phases, making some tiny cysts appear nodular. T2, AX: Axial T2−weighted image; DWI, B=0, AX: Axial diffusion-weighted imaging (B=0); DWI, B=600, AX: Axial diffusion-weighted imaging (B=600); DWI, B=1000, AX: Axial diffusion-weighted imaging (B=1000); T1, AX: Axial T1−weighted image; AP, AX: Axial arterial phase image; PVP, AX: Axial portal venous phase image; DP, AX: Axial delayed phase image.

Figure S2

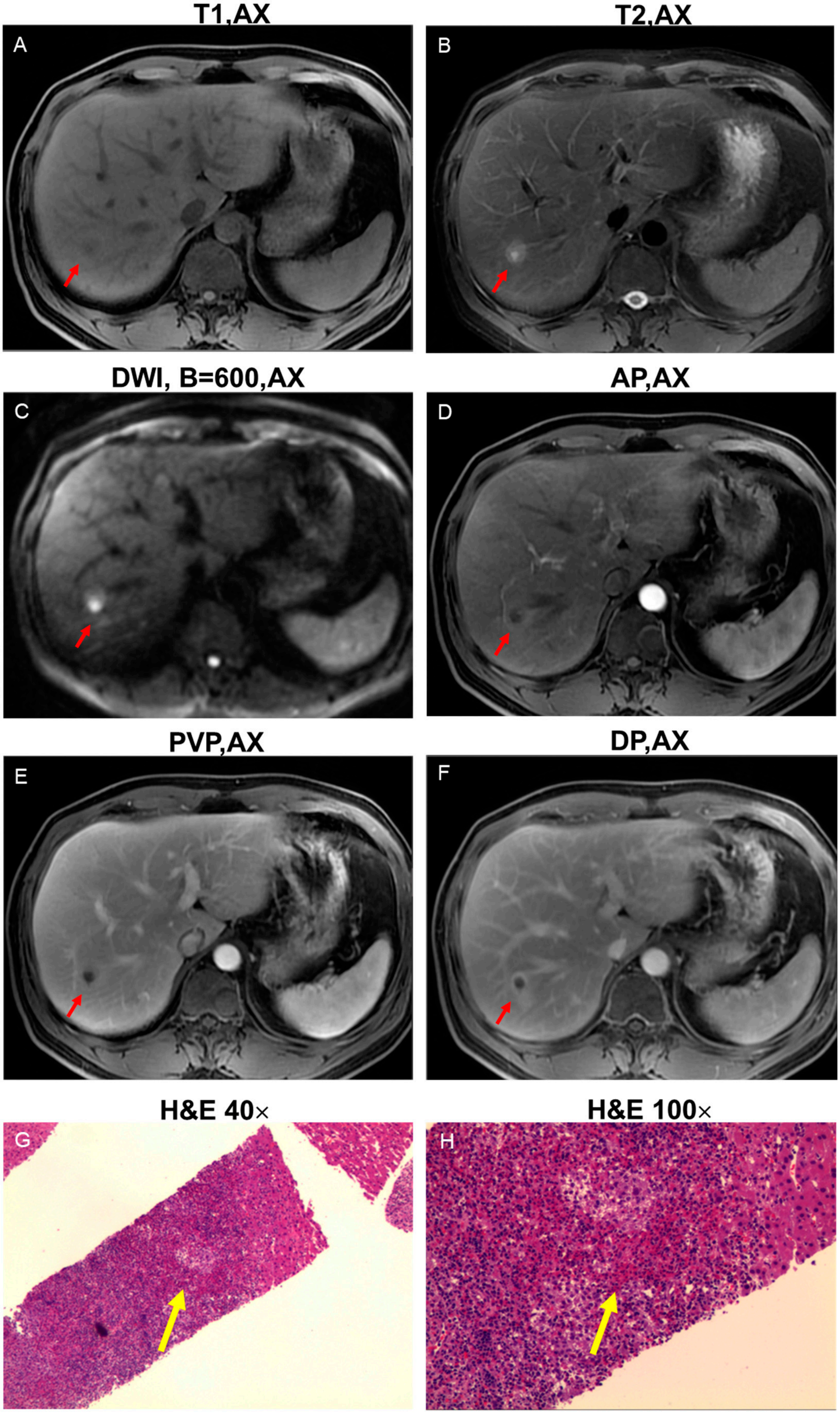

Figure S2. Imaging features of scattered lesion phenotype

A 14-year-old male teenager presented with an abdominal pain. He was diagnosed with multisystem LCH (MS-LCH) and classified as the scattered lesion phenotype. Focal lesions randomly scattered in liver parenchyma. A thick-walled cyst is observed in the right lobe of the liver (red arrow). The signal intensity inside the cyst was hypointense on T1WI (A) and hyperintense on T2WI/DWI (B, C); The thick wall presents a slightly high signal intensity on T2WI/DWI (B, C); ring-like enhancement from arterial phase to delayed phase was observed (D, E, F). Liver biopsy showed granulomatous inflammation (yellow arrow) (G. Hematoxylin and eosin (H&E), 40x; H. H&E, 100x). T1, AX: Axial T1−weighted image; T2, AX: Axial T2−weighted image; DWI, B=600, AX: Axial diffusion-weighted imaging (B=600); AP, AX: Axial arterial phase image; PVP, AX: Axial portal venous phase image; DP, AX: Axial delayed phase image.

Figure S3

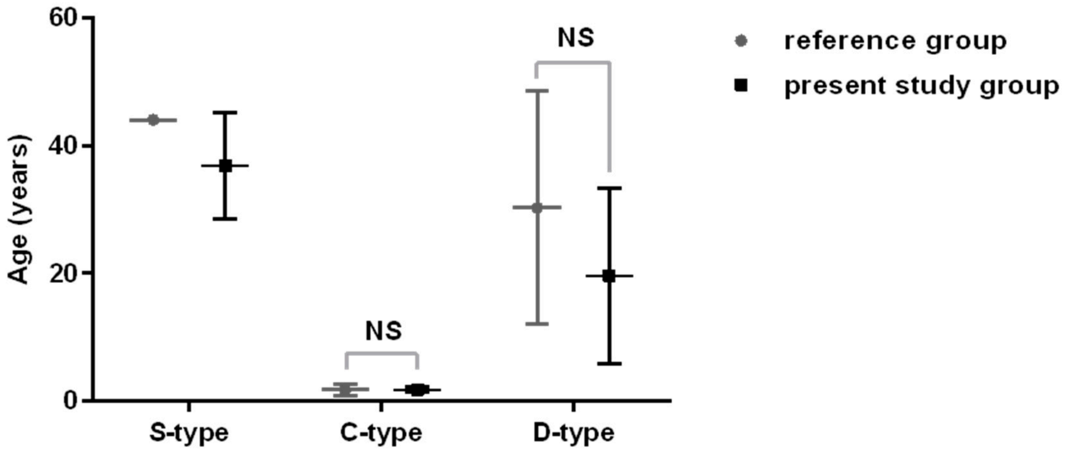

Figure S3. Comparison of age between the reference group and present study group.

S-type: scattered-lesion type; C-type: central-periportal-lesion type; D-type: disseminated-lesion type; NS, not statistically significant

Figure S4

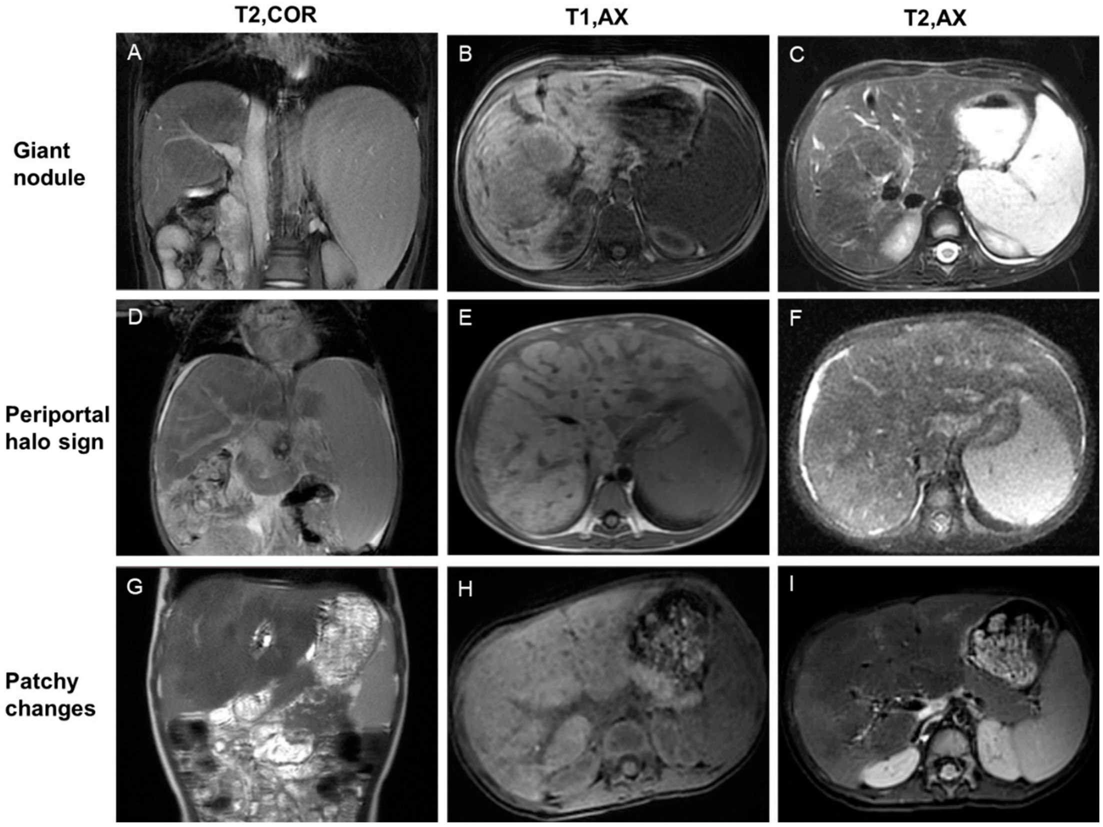

Figure S4. Three kinds of imaging sign of liver fibrosis in LCH patients

A, B, C: giant hepatic nodules close to the central portal tracts; D, E, F periportal halo sign; G, H, I: patchy parenchymal changes; T2, COR: Coronal T2−weighted image; T1, AX: Axial T1−weighted image; T2, AX: Axial T2−weighted image.

Supplementary Tables

Table S1 Imaging findings and modalities of present study group with three imaging phenotypes

Case	imaging phenotypes	Image modalities	Sex	Age	Parenchymal lesions		Periportal lesions		Bile duct
					Patchy	Nodular	thickening [a]	thickening [b]	abnormity
1	scattered lesion phenotype	CT, MRI, DCE-MRI	M	32	N	Y	N	N	N
2	scattered lesion phenotype	CT	F	53	N	Y	N	N	N
3	scattered lesion phenotype	CT, MRI, DCE-MRI, DWI	M	40	Y	Y	N	N	N
4	scattered lesion phenotype	CT	M	38	N	Y	N	N	N
5	scattered lesion phenotype	MRI, DWI	F	29	Y	Y	N	N	N
6	scattered lesion phenotype	CT, MRI, DCE-MRI, DWI	M	29	N	Y	N	N	N
7	disseminated lesion phenotype	CT, MRI, MRCP	M	21	Y	Y	Y	N	Y
8	disseminated lesion phenotype	CT, CECT, MRI, DCE-MRI, DWI	M	20	Y	Y	Y	N	N
9	disseminated lesion phenotype	CT	F	30	Y	Y	Y	N	N
10	disseminated lesion phenotype	CT	M	39	Y	Y	Y	N	N
11	disseminated lesion phenotype	CT	F	31	Y	Y	Y	N	Y
12	disseminated lesion phenotype	CT, MRI, MRCP	M	1.1	Y	Y	Y	N	Y
13	disseminated lesion phenotype	CT, CECT, MRI, DCE-MRI, DWI	M	14	Y	Y	Y	N	N
14	disseminated lesion phenotype	CT, MRI, DWI	F	1.3	Y	Y	Y	N	N
15	central periportal lesion phenotype	CECT, MRI	F	1.7	N	N	N	Y[b]	Y
16	central periportal lesion phenotype	CECT, MRI	M	1.8	N	N	N	Y[b]	Y
17	central periportal lesion phenotype	MRI, DWI	M	1.2	N	N	N	Y[b]	N
18	central periportal lesion phenotype	CT, MRI, DWI	M	1.8	N	N	N	Y[b]	Y
19	central periportal lesion phenotype	CT, MRI, MRCP	F	2.5	N	N	N	Y[b]	Y
20	central periportal lesion phenotype	CT, MRI, DCE-MRI, MRCP	M	1.8	N	N	N	Y[b]	Y

thickening [a]: slightly periportal space widening with periportal patchy or miliary lesions.

thickening [b]: serious band-like space widening

MS-LCH: Multisystem LCH; SS-LCH: Single System LCH; Y: Yes; N: No;

M: Male; F: Female; CT: computed tomography; CECT: contrast-enhanced computed tomography; MRI: magnetic resonance imaging; DCE-MRI: dynamic contrast-enhanced MRI; MRCP: magnetic resonance cholangiopancreatography;

Table S2 Published reports of LCH patients with liver involvement

Published reports	n [a]	Stratification	Sex	Age (years)	Hepatomegaly	Liver Biochemical abnormalities	Liver parenchymal lesion	Periportal lesions	Image phenotypes [b]	Bile duct abnormalities	Fibrotic signs [c]	Imaging modalities
Arakawa,1994	1	MS-LCH	M	18	NA	yes	yes	NA	NA	NA	1, P	CECT, MRI, US
CHAN,1997	2	MS-LCH	1M/1F	1.1;1.8	yes	yes	NA	yes	2, C	NA	NA	CECT, US
Mampaey,1999	1	MS-LCH	M	44	no	yes	yes	NA	1, S	no	NA	CECT, US
Kim,1999	3	MS-LCH	3M	0.9;2;1.5	yes	yes	NA	yes	3, C	2	1, P	MRI
Buza,2004	1	SS-LCH	F	9	yes	yes	yes	NA	NA	NA	NA	CT
Chaudhary,2006	1	MS-LCH	M	3	yes	yes	NA	yes	1, C	NA	NA	CT, US
Wong,2006	1	MS-LCH	M	0.9	no	yes	NA	yes	1, C	1	NA	CT, MRCP, MRI, US
Konno,2007	1	MS-LCH	M	35	no	yes	yes	NA	1, D	NA	NA	CECT
Caruso,2008	2	MS-LCH	M	4;1.3	yes	yes	no	yes	2, C	2	NA	CT, MRCP, US,
Savva-Bordalo,2008	1	MS-LCH	M	48	yes	no	yes	NA	1, D	NA	NA	CT
Gupta,2009	1	MS-LCH	M	1.2	NA	NA	no	yes	1, C	NA	NA	CECT
Abdallah ,2011	23	MS-LCH	14M/9F	37(19-87)	11(47.8%)	14(60.8%)	yes	Yes	1, D	13(56.5%)	NA	CT, MRI, US
Hu,2012	1	SS-LCH	F	27	no	yes	yes	NA	1, D	NA	NA	CECT, MRCP, MRI
Yuasa,2012	1	MS-LCH	F	23	yes	yes	yes	NA	1, D	no	NA	CT

Shi,2014	13	MS-LCH	7M/6F	2.4(1.1-4.3)	NA	NA	yes,7(54%)	Yes,13	3, C;1, D	11(84.6%)	NA	CT, MRCP, MRI
Ma,2014	1	MS-LCH	M	45	yes	yes	yes	NA	1, D	no	NA	MRI, DCE-MRI
Araujo,2015	1	MS-LCH	M	52	yes	yes	yes	NA	1, D	no	NA	CECT
Kapoor,2015	1	MS-LCH	M	0.5	yes	yes	NA	yes	1, C	no	NA	CT
Zhang,2015	6	MS-LCH	3M/3F	45.5(32-64)	NA	1(16.7%)	yes	no	NA	no	NA	CT, DCE-MRI, MRI
Tang,2017	1	MS-LCH	M	31	yes	yes	NA	NA	NA	1	1, PH	CE-MRI, MRCP
Ouizeman,2018	1	MS-LCH	M	69	yes	yes	NA	NA	NA	1	NA	MRCP
Rajavelu,2019	1	MS-LCH	F	1.5	yes	yes	yes	yes	1, D	NA	NA	CECT, MRCP, US
Rayamajhi,2020	1	MS-LCH	F	41	no	yes	NA	NA	NA	NA	1, PH	PET/CT
Wang,2020	1	MS-LCH	F	37	no	yes	yes	NA	NA	1	1, PH	MRI, MRCP

[a] Number of LCH patients with liver involvement

[b] Number of clinical cases that have infiltrative lesions demonstrated by imaging figures in reports and corresponding imaging phenotypes that were classified based on distribution and morphology of lesions. S: scattered lesion phenotype; D: disseminated lesion phenotype; C: central periportal lesion phenotype;

[c] Number of clinical cases that have been reported with fibrotic hypointensity on T2WI or hypodensity on CT/MRI and corresponding pattern. PH: periportal halo sign; P: patchy liver parenchyma fibrosis;

MS-LCH: multisystem LCH; SS-LCH: single system LCH; M: Male; F: Female; CT: computed tomography; CECT: contrast-enhanced computed tomography; MRI: magnetic resonance imaging; DCE-MRI: dynamic contrast-enhanced MRI; US: ultrasonography; PET/CT: positron emission tomography/computed tomography; MRCP: magnetic resonance cholangiopancreatography;

Table S3 Summary of Image phenotypes and pathologic findings of the liver in three patients

case	Image phenotypes	pathology
3	scattered lesion phenotype	granulomatous inflammation
14	disseminated lesion phenotype	focal proliferation of histiocytes;<br>periportal inflammatory cells;<br>periportal fibrosis and bile ductular proliferation
17	central periportal lesion phenotype	chronic hepatitis;<br>periportal inflammatory cells;<br>periportal fibrosis and bile ductular proliferation

Table S4 Evolution of parenchymal lesions in eight patients

Case	Distribution type	Initial MRI scan			Last MRI scan			Evolution trend
		patchy and miliary lesions	thick-walled cystic-like lesions	thin-walled cystic-like lesions	patchy and miliary lesions	thick-walled cystic-like lesions	thin-walled cystic-like lesions	
1	S-type	N	Y	Y	N	N	Y	regression
3	S-type	Y	Y	N	N	N	Y	regression
5	S-type	Y	N	N	N	N	N	compete resolution
6	S-type	N	N	Y	N	N	N	compete resolution
7	D-type	Y	Y	N	N	Y	Y	regression
8	D-type	Y	Y	Y	N	Y	Y	regression
13	D-type	Y	N	N	N	N	Y	regression
14	D-type	Y	Y	N	Y	N	Y	regression
Total		6	5	3	1	2	6	

Table S5 ADC values for the b-values of 1,000 s/mm^2

case	T2/DWI hypointense	ADC-value (mean ± SD)	ADC-value (mean ± SD)
3	(-)	1.21 ± 0.11	1.25 ± 0.14
8	(-)	1.22 ± 0.15	
13	(-)	1.31 ± 0.15	
14	patchy liver parenchyma changes	0.97 ± 0.04	0.97 ± 0.10
16	periportal halo sign	1.00 ± 0.05	
17	patchy liver parenchyma changes	0.81 ± 0.1	
18	giant, central nodular fibrosis	1.03± 0.03	
20	patchy liver parenchyma changes	1.01 ± 0.03	

ADC, apparent diffusion coefficient（10^{-3}mm^2/s）
